# A Discrete Mathematics Approach for Understanding Risk Factors in Overactive Bladder Treatment

**DOI:** 10.7759/cureus.53245

**Published:** 2024-01-30

**Authors:** Nobuo Okui

**Affiliations:** 1 Urology, Yokosuka Urogynecology and Urology Clinic, Kanagawa, JPN

**Keywords:** frailty, onabotulinum toxin a injections, clustering analysis, network graphs, graph theory, discrete mathematics, post-void residual urine, overactive bladder

## Abstract

Introduction

Discrete mathematics, a branch of mathematics that includes graph theory, combinatorics, and logic, focuses on discrete mathematical structures. Its application in the medical field, particularly in analyzing patterns in patient data and optimizing treatment methods, is invaluable. This study, focusing on post-void residual (PVR) urine following overactive bladder (OAB) treatment, utilized discrete mathematics techniques to analyze PVR and its associated risk factors.

Methods

A retrospective study was conducted on 128 OAB patients who received intradetrusor onabotulinum toxin A injections between 2020 and 2022. Network graphs based on graph theory were used to analyze correlations between clinical variables, and clustering analysis was performed with PVR as the primary variable.

Results

The network graph analysis revealed that frailty, daytime frequency, and nocturia episodes were closely related to PVR. Clustering analysis with PVR as the primary variable divided the patients into three groups, suggesting that the group with particularly high frailty (Cluster 1) is at high risk for PVR. Moreover, significant differences in clinical indicators such as age, voiding efficiency, Overactive Bladder Symptom Score, and International Consultation on Incontinence Questionnaire-Short Form were observed in the remaining two clusters (Cluster 0 and 2).

Conclusion

This study demonstrates the effectiveness of discrete mathematics methods in identifying risk factors for PVR after OAB treatment and in distinguishing clinical subgroups based on patient characteristics. This approach could contribute to the formulation of individualized treatment strategies and the improvement of patient care quality. Further development and clinical application of this methodology are expected in future research.

## Introduction

Discrete mathematics is a branch of mathematics focused on discrete mathematical structures, including graph theory, combinatorics, and logic. According to literature, this field emphasizes the study of entities composed of distinct, often finite, parts [1-3]. The application of discrete mathematics in clinical medicine is particularly useful for analyzing patient data patterns and optimizing treatment methods, contributing to improved risk assessment [4,5]. Previous studies have gradually introduced examples of applying discrete mathematics in the medical field, primarily related to discovering potential treatment patterns in clinical processes [3,6]. However, there have been no applications of this mathematical characteristic in addressing the side effects of treatments or surgeries.

This study aims to analyze the issue of residual urine (a side effect) following intradetrusor injections of onabotulinum toxin A using discrete mathematics methods. Intradetrusor injections of onabotulinum toxin A are widely used in treating overactive bladder, especially in elderly women, and post-treatment residual urine is one of the major side effects [7-9]. Notably, our previous reports have brought new insights by focusing on frailty [10]. In this study, we aim to provide new insights as potential treatment patterns using discrete mathematics, reusing data obtained from our previous research.

## Materials and methods

Study design and approval

This retrospective study enrolled overactive bladder (OAB) patients who underwent 100U intradetrusor injections of onabotulinum toxin A (GlaxoSmithKline, Tokyo, Japan) between 2020 and 2022 [10]. All patients provided written informed consent after receiving information about potential adverse events associated with these injections [10]. Each patient received a total of 100U of onabotulinum toxin A injections intradetrusorly into the bladder, with the trigone being excluded. This study constitutes a post-hoc analysis of a prior study [10]. It received approval from the regional medical ethics committee (Ethics Review Committee of Yokosuka Urogynecology and Urology Clinic, Protocol No. 23005). 

Patient selection criteria

The indications for intradetrusor onabotulinum toxin A injections included patients who had exhibited resistance to treatment with antimuscarinic drugs and beta-3 adrenergic agents for a minimum of four months. They continued to experience severe urgency urinary incontinence or had at least one episode of urgency incontinence per day, despite prior treatment involving at least two types of antimuscarinic drugs and one type of beta-3 adrenergic agent. Patients with pelvic organ prolapse at Stage II or lower were considered eligible for the treatment group. At the time of study registration, all patients did not present with neurogenic bladder, intrinsic sphincter deficiency, urinary tract infection, congenital malformation, or vesicovaginal fistula [10].

Urodynamic studies

Urodynamic studies were carried out using the Goby family wireless urodynamic system (EDAP TMS, Rhône, France) to evaluate detrusor overactivity, bladder outlet obstruction, and intrinsic sphincter deficiency [11]. Standard urodynamic assessments also included the Urethral Pressure Profile (UPP) [11]. Female participants with bladder outlet obstruction or diminished detrusor activity were excluded from the study. Bladder outlet obstruction was determined by the presence of narrowing at the bladder exit, detrusor pressure exceeding 35 cmH^2^O, maximum flow (Qmax) falling below 15 mL/s, or detrusor pressure surpassing 40 cmH^2^O. Patients were identified as having reduced detrusor activity if the contraction strength during voiding was ≤ 10 cmH^2^O, abdominal straining was required, or if voiding was impossible. Various parameters were measured, including the initial sensation of filling, bladder capacity during intravesical pressure measurement, bladder compliance, Qmax, post-void residual (PVR) volume, Pdet.Qmax at Qmax, and Bladder Contraction Index (BCI = Pdet.Qmax + 5 × Qmax). Furthermore, voiding efficiency was evaluated by calculating the voided volume as a percentage of bladder capacity [12].

Evaluation of OAB

During clinic visits, multiple assessments were conducted, including measurements of various parameters such as voided volume, PVR volume, and Qmax. Based on a three-day voiding diary, the number of daytime frequency and nocturia episodes within 72 hours was calculated [13]. PVR volume measurement was performed using transabdominal ultrasonography. Bladder capacity was determined by combining voided volume and PVR volume. The severity of urinary incontinence was assessed using the International Consultation on Incontinence Questionnaire-Short Form (ICIQ-SF), considering frequency, volume, and impact on daily life. Overactive Bladder Symptom Score (OABSS) was measured to evaluate daytime and nocturnal voiding frequency, urgency, and incontinence [14,15]. The presence of OAB with wet symptoms (OAB-wet) or OAB without wet symptoms (OAB-dry) was determined based on a three-day voiding diary. Patients with at least one episode of urgency incontinence recorded were considered OAB-wet, and the rest were considered OAB-dry. The Patient Perception of Bladder Condition (PPBC) is a self-assessment tool used to evaluate patient perception and the impact on daily life, especially regarding symptoms associated with OAB [16]. Urgency was assessed using a modified version of the validated Indevus Urgency Severity Scale (USS) [17].

Questionnaire on frailty

Patients were also interviewed using a frailty questionnaire that assessed fatigue, resistance, ambulation, illness, and weight loss. Each item was scored on a scale of 1 to 5, with scores of 1-2 or ≥ 3 out of 5 indicating frailty and pre-frailty (FRAILTY), respectively [18].

Post-void residual volume

The duration of the interval with persistent large PVR volumes was measured from the day the first large PVR volume was recorded during follow-up visits to the day when the absence of large PVR volumes was documented, and then to the last follow-up visit. According to previous literature, a PVR volume exceeding 200 mL was considered large [10].

Methods of discrete mathematics

In the study, network graphs were utilized to visualize and analyze correlations between variables. The "NetworkX" library in Python (Python Software Foundation, Beaverton, OR, USA) was used for network analysis. This library was employed to depict the associations between multiple clinical variables, including PVR, and to analyze their interactions.

The correlation coefficients used in the network graph were Pearson correlation coefficients and Spearman's rank correlation coefficients.

In clustering analysis, the KMeans clustering function within the "scikit-learn" library was utilized. In this study, the KMeans algorithm was used to divide patients into three different clusters, and the characteristics of each cluster were analyzed in detail.

Principal component analysis (PCA) was used for the analysis. PCA is a statistical method for representing multidimensional data in fewer dimensions, capturing data variability, reducing dimensions, facilitating visualization and interpretation, and reducing noise for efficient data analysis.

A multivariate logistic regression analysis incorporating all significant factors was performed to predict the probability (p) of a significant PVR volume and to obtain a logit transformation (logit(p)) [10]. Statistical methods employed for variables between clusters included the Kruskal-Wallis test, ANOVA, and Chi-squared test.

Continuous variables are reported as mean ± standard deviation or as numbers and percentages. The data provided statistical guidance and was used to generate Python language code with the assistance of ChatGPT (OpenAI, San Francisco, CA, USA).

## Results

Patients

A total of 2,881 patients with OAB sought medical consultation. Out of these, 212 did not require OAB medication. Among the patients who had been on OAB medication for over four months, 131 were eligible for intravesical onabotulinum toxin A injections. During the six-month follow-up period, our study included 128 women (age range: 44-91 years, median age: 72.5 years). In this cohort, 23 women (18.0%) experienced PVR volume exceeding 200 mL [10].

Discrete mathematics graph theory

**Figure 1:**
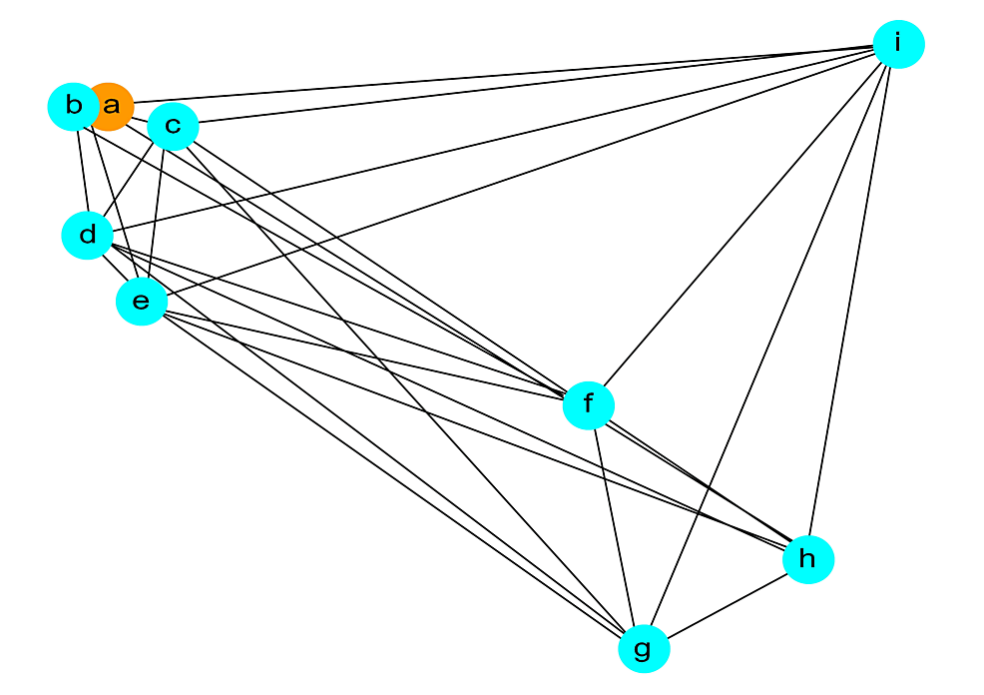
Network Analysis of High Post-Void Residual (PVR) Risk Factors in Overactive Bladder (OAB) Treatment. The network graph depicted in this figure visually represents the correlation relationships between selected variables. In this network graph, there are no x or y axes; the nodes (points) represent variables, and the edges (lines) indicate the correlation relationships between these variables. This visualization provides an intuitive means to understand the relationships between variables. Node a: PVR, b: 72h-nocturia episodes, c: FRAILTY, d: 72hr-daytime frequency, e: logit(p), f: bladder contractility index, g: OAB-wet, h: cytometric bladder capacity, i: voiding efficiency. Edges: Representing correlation relationships between variables.

Figure 1 shows the network graph. In this graph, selected variables (PVR, 72hr-daytime frequency, FRAILTY, voiding efficiency (%), logit(p), OAB-wet, 72hr-nocturia episodes, cystometric bladder capacity (mL), bladder contractility index) are represented as nodes, and the edges between each node indicate the correlations between these variables.

PVR is depicted in orange, while the other variables are shown in light blue. Each node in the graph represents a specific variable, and the thickness of the edges reflects the strength of the correlations between these variables. Visually, you can observe that variables in proximity to PVR are 72hr-nocturia episodes (b), FRAILTY (c), and 72hr-daytime frequency (d).

Table 1 lists the edges between each node (variable) in the network graph and their correlational relationships. The table is organized in descending order of correlation coefficients, providing an illustration of the correlational relationships between the selected variables. It particularly emphasizes pairs with high correlation coefficients, indicating strong associations between these variables.

**Table 1:** Correlation Coefficients for Variables Related to Post-Void Residual Urine Following Onabotulinum Toxin A Injection. PVR: post-void residual OAB: overactive bladder OAB wet: overactive bladder with wet symptoms FRAILTY: frailty and pre-frailty

Variable Pair	Correlation Coefficient
72hr-daytime Frequency - logit(p)	0.9296
PVR - FRAILTY	0.7974
PVR - 72hr-daytime frequency	0.7397
PVR - logit(p)	0.739
FRAILTY - logit(p)	0.6381
72hr-daytime frequency - FRAILTY	0.5993
PVR - 72hr-nocturia episodes	0.4594
FRAILTY- 72hr-nocturia episodes	0.424
72hr-daytime frequency - 72hr-nocturia episodes	0.4052
logit(p) - 72hr-nocturia episodes	0.4016
Voiding efficiency (%) - OAB wet	0.1701
OAB wet - Bladder contractility index	0.14
Voiding efficiency (%) - Cystometric bladder capacity (mL)	0.1219
OAB wet - Cystometric bladder capacity (mL)	0.1212
Voiding efficiency (%) - Bladder contractility index	0.087
72hr-daytime frequency - Bladder contractility index	-0.0025
PVR - Bladder contractility index	-0.0192
Cystometric bladder capacity (mL) - Bladder contractility index	-0.0209
FRAILTY - Bladder contractility index	-0.028
72hr-nocturia episodes - Bladder contractility index	-0.0342
logit(p) - Bladder contractility index	-0.0531
FRAILTY - Cystometric bladder capacity (mL)	-0.0961
Voiding efficiency (%) - 72hr-nocturia episodes	-0.1417
72hr-daytime frequency - OAB wet	-0.1779
72hr-daytime frequency - Cystometric bladder capacity (mL)	-0.1816
logit(p) - Cystometric bladder capacity (mL)	-0.204
OAB wet - Nocturia episodes (72 h)	-0.205
PVR - Cystometric bladder capacity (mL)	-0.206
PVR - OAB wet	-0.2113
72hr-nocturia episodes - Cystometric bladder capacity (mL)	-0.2564
FRAILTY - OAB wet	-0.2586
72hr-daytime frequency - Voiding efficiency (%)	-0.3209
PVR - Voiding efficiency (%)	-0.3538
logit(p) - OAB wet	-0.3559
FRAILTY - Voiding efficiency (%)	-0.3632
Voiding efficiency (%) - logit(p)	-0.6155

Cluster analysis using discrete mathematics algorithm theory

**Figure 2:**
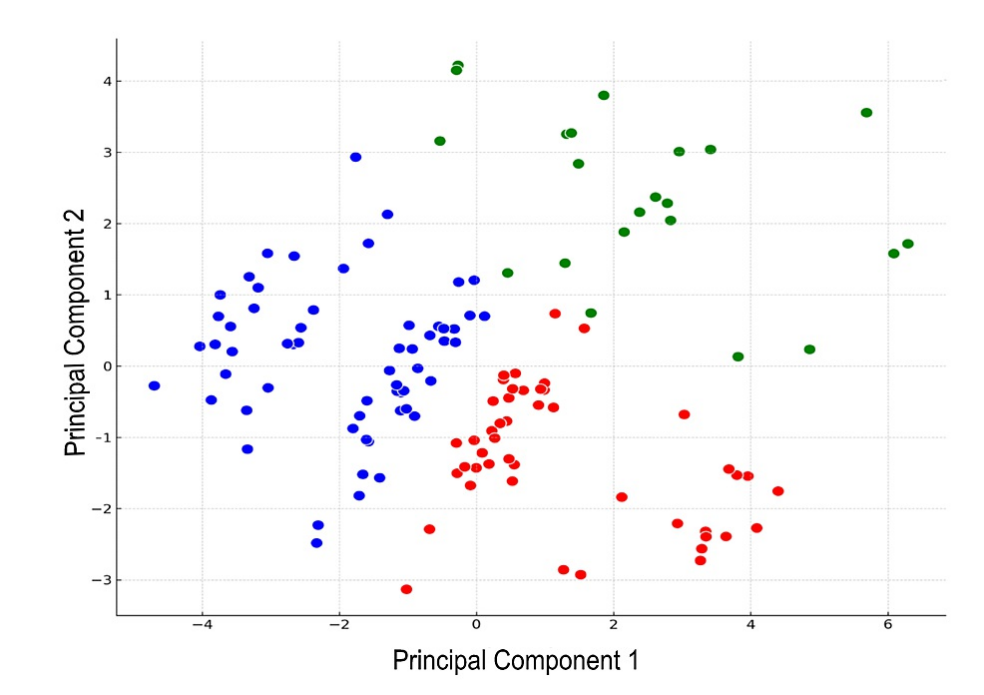
K-Means Clustering With Three Clusters X-axis: Principal Component 1, Y-axis: Principal Component 2 Blue closed circles: Cluster 0, Green closed circles: Cluster 1, Red closed circles: Cluster 2.

Figure 2 demonstrates an analysis based on combinatorial theory from discrete mathematics, where we examined the combinations of patient characteristics and treatment outcomes (specifically, PVR as a side effect) using selected variables and applied KMeans clustering. In this analysis, after standardizing the data, we employed PCA for dimension reduction, allowing for more effective visualization. Consequently, patient data were classified into three distinct clusters. PCA dimension reduction results revealed that the first two principal components accounted for approximately 48.68% of the data variance (roughly 31.57% by the first principal component and about 17.11% by the second principal component). This approach aided in identifying optimal patient profiles. The clusters are denoted as Cluster 0 (blue), Cluster 1 (green), and Cluster 2 (red).

Table 2 shows the analysis of clusters 0, 1, and 2. Cluster 0 and Cluster 2 both exhibit low PVR values, suggesting the absence of significant PVR, with Cluster 2 having slightly lower values (p < 0.001, Kruskal-Wallis test). This indicates a statistically significant difference in PVR values among the clusters. Cluster 2 has a higher average age compared to Cluster 0 (p < 0.001, Kruskal-Wallis test), potentially indicating a higher risk of urinary incontinence in older individuals within Cluster 2. Voiding efficiency: Cluster 0 shows higher voiding efficiency, while Cluster 2 exhibits slightly lower values (p < 0.001, ANOVA). This implies that patients in Cluster 2 may experience minor voiding issues, and this difference is statistically significant. OABSS and ICIQ-SF: Cluster 2 demonstrates more severe symptoms with higher OABSS scores and a greater impact on ICIQ-SF (OABSS: p < 0.001, ICIQ-SF: p < 0.001, both Chi-squared test). This indicates a more pronounced presence of these issues in Cluster 2, and the differences are statistically significant.

**Table 2:** Cluster-Based Comparative Analysis of Urinary and Bladder Function Parameters. PVR: post-void residual FRAILTY: frailty and pre-frailty OABSS: Overactive Bladder Symptom Score USS: uroflowmetry and post-void residual urine volume PPBC: Patient Perception of Bladder Condition ICIQ-SF: International Consultation on Incontinence Questionnaire - Short Form Pdet.Qmax: detrusor pressure at maximum flow

Variable	Unit	Cluster 0	Cluster 1	Cluster 2	The significance of differences in the three groups
Number of individuals	Number	56	25	47	N/A
Age	years old	68.55 ± 13.19	79.52 ± 7.12	72.70 ± 9.70	p<0.001
PVR	ml	66.7 ± 56.46	247.20 ± 53.16	60.74 ± 40.09	p<0.001
FRAILTY	Number（％）	1 (1.79％)	22 (88.00％)	3 (6.38％)	p<0.001
OAB wet	Number（％）	55 (98.21％)	17 (68.00％)	42 (89.36％)	p<0.001
72hr-daytime frequency		26.64 ± 2.09	32.32 ± 3.51	27.02 ± 1.73	p<0.001
Voiding efficiency	%	89.22 ± 4.08	78.33 ± 6.16	80.24 ± 4.18	p<0.001
logit(p)		3.71 ± 1.31	8.39 ± 1.99	5.01 ± 1.08	p<0.001
72hr-nocturia episodes		7.05 ± 2.01	9.24 ± 1.54	6.06 ± 2.08	p<0.001
Cystometric bladder capacity	mL	225.59 ± 36.20	208.68 ± 17.71	225.34 ± 29.02	p = 0.0766
Bladder contractility index		95.89 ± 20.70	91.88 ± 23.69	93.96 ± 24.31	p = 0.8894
OABSS		11.20 ± 1.03	12.52 ± 1.69	13.60 ± 1.04	p<0.001
USS		2.54 ± 0.503	3.00 ± 0.645	3.32 ± 0.471	p<0.001
PPBC		4.48 ± 0.504	4.88 ± 0.60	5.23 ± 0.428	p<0.001
First sensation	mL	114.73 ± 10.82	115.84 ± 11.52	117.62 ± 12.98	p = 0.4472
Detrusor overactivity		0.98 ± 0.134	0.96 ± 0.200	0.96 ± 0.204	p = 0.6061
ICIQ-SF		12.61 ± 1.40	15.96 ± 2.78	16.74 ± 1.98	p<0.001
Pdet.Qmax	cmH^2^O	26.66 ± 8.12	27.00 ± 8.60	28.70 ± 9.08	p = 0.6872

## Discussion

Graph theory, a fundamental component of discrete mathematics, offers a mathematical framework for studying network graphs, which find a wide range of applications in addressing real-world problems [19]. Amanathulla et al.'s research illustrates how graph coloring and labeling play crucial roles in practical problem-solving, including scheduling, mobile phone networks, Google Maps, traffic signals, social networks, and airline scheduling [20]. Coufal et al. delve into the utilization of graphs and Graph Theory in the realm of autonomous motion of robots [21]. This field is deeply rooted in discrete mathematics and computer science, introducing novel algorithmic approaches for data analysis via network graphs. Dawood et al. provide an insightful overview of the applications of graph theory in network security, encryption, and cybersecurity [22], serving as a valuable resource for comprehending the role of graph theory in computer security.

In this study, we analyzed the risk factors for high PVR following OAB treatment using network graphs. We identified that 'FRAILTY,' '72hr-nocturia episodes,' and '72hr-daytime frequency' are closely associated with PVR, providing insights into the influence of these patient characteristics.

On the other hand, previous research by Hsiao et al. used multivariate analysis and identified '72hr-daytime frequency' and 'voiding efficiency' as independent predictive factors for high PVR [23]. In our previous study, we analyzed the risk factors for high PVR using random forest and support vector machine models and identified 'FRAILTY,' '72hr-daytime frequency,' and 'voiding efficiency' as important predictive factors for PVR [10].

Comparing these previous studies with our current research, graph theory consistently supports the impact of '72hr-daytime frequency' on PVR. However, the strong associations found in our study for 'FRAILTY' and 'nocturia episodes' were not emphasized in previous research, and 'voiding efficiency' showed a lower relationship with PVR in the data of all patients, including those with frailty and pre-frailty.

Multivariate analysis conducted in previous medical research and the graph theory used in this study provide different insights and complement each other as tools [24]. In future medical research, network graphs can visualize direct relationships between variables, making them suitable for initial exploratory analysis and quickly identifying strong correlations between specific variables [25]. Multivariate analysis delves deeper into these relationships, considering the influence of other variables and revealing more complex causal relationships. In other words, the relationships uncovered by network graphs serve as a starting point for further verification through multivariate analysis.

Clustering becomes possible through algorithms in discrete mathematics. Previous research in this field spans various domains. A study by Lijoi et al. presented a new prior distribution for Bayesian clustering, replacing the standard Dirichlet-multinomial model, showing the potential to overcome limitations of existing models [26]. Additionally, the research by Yu et al. addresses the clustering problem of data with known partial grouping as a constrained optimization problem [27].

In our study, clustering analysis based on discrete mathematics revealed distinct subgroups with different clinical characteristics among patients with OAB who received intravesical onabotulinum toxin A injections. Of particular note is the high presence of 'FRAILTY' in Cluster 1. This result suggests that frailty and pre-frailty are important risk factors in OAB treatment, consistent with our previous study, emphasizing frailty and pre-frailty as a significant category in intravesical onabotulinum toxin A injections.

Comparing Cluster 0 and Cluster 2, significant differences were observed in clinical indicators such as PVR, age, voiding efficiency, OABSS, and ICIQ-SF. Patients in Cluster 2 had a higher average age and lower voiding efficiency, characteristics suggesting a higher risk of urinary incontinence in the elderly, as indicated in the study by Hsiao et al. Clustering in our study provided visual insights and offered new information for decision support in patient care.

The utilization of clustering in our study provided a new approach to support decision-making processes in clinical medicine. Understanding how different patient characteristics are related to the risk of high PVR helps in developing personalized treatment strategies [28]. This approach enables a deeper understanding of patient groups and helps select the optimal treatment for patients with specific characteristics [29]. In clinical medicine, considering which cluster a patient belongs to when planning treatment can lead to predicting treatment response and improving patients' Quality of Life (QOL) while maximizing treatment effectiveness [30].

Our study has limitations, including limitations in the dataset. The study is based on treatments received at specific clinics in a particular region, potentially limiting the results to that specific patient population. This may restrict the generalization of results to other regions and settings. The retrospective nature of the study poses limitations. As a retrospective study, it relies on post hoc data collection, which carries a high risk of bias and makes it challenging to establish causality. Sample size and representativeness are also limitations. The limited sample size in the study may affect statistical significance and the representativeness of results. A small sample size can reduce the reliability of conclusions.

## Conclusions

In this study, it became evident that the application of graph theory and algorithms from discrete mathematics provides valuable insights in medical research. Network graph analysis helped deepen the understanding of risk factors related to PVR following treatment for OAB, especially highlighting variables such as frailty and pre-frailty, 72hr-daytime frequency, and 72hr-nocturia episodes as closely related to PVR. These findings provide crucial information for decision-making in clinical treatment strategies.

Furthermore, clustering analysis in our study revealed distinct clinical characteristics among patient groups, particularly emphasizing the high presence of frailty and pre-frailty in Cluster 1, suggesting it as an essential risk factor in OAB treatment. This approach contributes to the development of personalized treatment plans and predicts how patient groups with different characteristics respond to treatment, aiming to improve patient QOL and maximize treatment effectiveness.

Our research demonstrates the effectiveness of applying methods from discrete mathematics to the field of medicine. In the future, such approaches are expected to evolve into essential tools in medical research and decision support. Additionally, applying them to various medical domains will likely provide new solutions to various clinical challenges.
